# Prevalence of Sleep Disturbances in Rheumatoid Arthritis and Its Association With Disease Severity: A Hospital-Based Cross-Sectional Observation

**DOI:** 10.7759/cureus.84767

**Published:** 2025-05-25

**Authors:** Debashish K Debta, Krishna Padarabinda Tripathy, Tejash Jain, Pradip K Behera, Sasidhar Chodey, Mrinalini Sai, Pooja Agarwal, Bharath V Karnati, Prasanta Padhan

**Affiliations:** 1 General Medicine, Kalinga Institute of Medical Sciences, Bhubaneswar, IND; 2 Community Medicine, Veer Surendra Sai Institute of Medical Sciences and Research, Sambalpur, IND; 3 Rheumatology, Kalinga Institute of Medical Sciences, Bhubaneswar, IND

**Keywords:** disease activity, disease activity score-28, insomnia, pittsburgh sleep quality index, rheumatoid arthritis, sleep disturbances

## Abstract

Introduction

Rheumatoid arthritis (RA), a chronic inflammatory disorder, is associated with significant joint pain, functional disability, and systemic inflammation. Sleep, a vital physiological process, plays a crucial role in immune regulation and tissue repair. Disruptions in sleep patterns are prevalent among RA patients, yet remain underdiagnosed and undertreated. A two-year study investigates the prevalence of sleep disturbances in RA patients and explores the association between pain perception and disease severity.

Materials and methods

A cross-sectional observational study was conducted from March 2023 to March 2025 in the Departments of General Medicine and Rheumatology at KIMS PBMH, Bhubaneswar. The study included 176 participants, comprising 88 RA patients diagnosed using the 2010 ACR-EULAR Classification criteria and 88 age and sex-matched controls. Sleep quality was assessed using the Pittsburgh Sleep Quality Index (PSQI), with a score ≥5 indicating poor sleep quality. Insomnia severity was evaluated using the Insomnia Severity Index (ISI). RA disease activity was classified using the Disease Activity Score-28 (DAS-28). Pain perception as per the Visual Analogue Scale (VAS) was graded as no pain, mild, moderate or severe.

Results

Among RA patients, 66 (74.0%) were classified as poor sleepers compared to 5 (5.7%) of controls (p<0.001). Insomnia was prevalent in 67 (76.1%) of RA patients, with 37 (42.0%) experiencing moderate insomnia and 24 (27.3%) severe insomnia (p<0.001). Severe pain was reported by 26 (29.5%) of RA patients versus none in the control group (p<0.001). Disease activity was significantly associated with sleep disturbances: 22 (25%) had mild RA, 38.6% moderate, and 36.4% severe disease activity. Higher DAS-28 scores correlated with poorer sleep quality and increased insomnia severity (p<0.001). Severe pain was a significant determinant of sleep disruption (p<0.001). The findings underscore a strong link between RA, pain, and sleep disturbances.

Conclusion

Sleep disturbances are highly prevalent in RA patients and strongly correlate with disease severity and pain perception. Routine sleep assessment should be integrated into RA management. Interventions such as cognitive behavioral therapy for insomnia (CBT-I) and pharmacologic treatments targeting both pain and sleep may improve clinical outcomes. Further longitudinal research is warranted to explore causal pathways and the impact of sleep interventions on RA progression.

## Introduction

Sleep is a complex biological process that is governed by circadian rhythms and homeostatic mechanisms, ensuring optimal physiological and psychological health. Sleep is essential for immune system modulation, cognitive function, and physical recovery. Rapid eye movement (REM) and non-REM sleep are two of the different stages of sleep that each support cellular repair, metabolic balance, and memory consolidation. Sleep plays a role that goes beyond simple relaxation because disruptions in its architecture have been connected to elevated inflammation, hormone imbalances, and the advancement of chronic diseases [1].

Sleep disruptions continue to be underdiagnosed and undertreated despite their significant negative effects on health, especially in rheumatoid arthritis (RA), where pain and systemic inflammation further reduce the quality of sleep [2]. Prolonged joint inflammation is a characteristic of RA, a chronic inflammatory disease that causes pain and functional impairment [3]. Sleep disturbances are highly prevalent among RA patients, with studies indicating that a significant proportion experience poor sleep quality [4]. There are several facets to the connection between RA and sleep issues, where chronic pain associated with RA can lead to difficulty falling asleep and frequent nocturnal awakenings [5]. Tumor necrosis factor-alpha (TNF-α) and other inflammatory cytokines can interfere with regular sleep cycles and regulate sleep [6].

Despite the recognized importance of sleep in health and disease, research focusing on sleep disturbances, particularly in the context of chronic illnesses like RA, remains underrepresented both in India and globally. Several factors contribute to this gap, including a lack of awareness: sleep issues are often underreported by patients and underrecognized by healthcare providers, leading to a paucity of data and research initiatives [7]. Resource constraints in developing countries, including India, may prioritize infectious diseases and malnutrition over sleep research due to limited healthcare resources [8]. In many societies, cultural influences also play a part where sleep disturbances are not perceived as medical issues warranting attention, but rather as lifestyle concerns [9]. Thus, the main objective of the present study is to explore the occurrence of sleep disturbances in RA patients and to establish the correlation of disease activity severity with sleep disturbances.

## Materials and methods

Study design

This study design and setting is a cross-sectional observation, conducted in the Department of General Medicine and Department of Rheumatology at Kalinga Institute of Medical Sciences-Pradyumna Bal Memorial Hospital (KIMS PBMH), Bhubaneswar, over a period from March 2023 to March 2025.

Study population

In order to provide a diverse sample of rheumatoid arthritis, the study population included both inpatients and outpatients. Individuals having a rheumatoid arthritis (RA) diagnosis according to the 2010 ACR-EULAR were enrolled. Age- and sex-matched attendants or family members without RA were taken as the control group.

Sample size

The case group (Group 1) comprised all consecutive patients with rheumatoid arthritis (RA) who were diagnosed with sleep disturbances during the study period. Age- and sex-matched healthy individuals served as the control group (Group 2). A total of 176 participants were included in the study, with 88 individuals in the RA group (Group 1) and 88 in the control group (Group 2).

Inclusion & exclusion criteria

Group 1 consisted of patients aged ≥16 years who were diagnosed with rheumatoid arthritis (RA) and were on stable pharmaceutical therapy, as per the inclusion criteria. Likewise, Group 2 comprised healthy adults aged ≥16 years who were matched by age and sex.

Those with primary sleep disorders (like obstructive sleep apnoea), those on sleep aids, people with primary mental illnesses, other chronic conditions, autoimmune diseases (like Sjögren's syndrome and systemic lupus erythematosus), cancer, pregnant women, and critically ill patients were excluded from the study.

Study tools

Assessment of sleep disturbances was done by the Pittsburgh Sleep Quality Index (PSQI) for subjective sleep quality. A PSQI score ≥5 was considered indicative of poor sleep quality. The Insomnia Severity Index (ISI) was also used to classify insomnia severity. Patients clinically indicated for further evaluation underwent sleep studies. Assessment of disease severity was done by the Disease Activity Score-28 (DAS-28). Patients were categorized as - Low disease activity (DAS-28 <3.2), Moderate disease activity (DAS-28 3.2-5.1), and High disease activity (DAS-28 >5.1). The pain perception was assessed by using a Visual Analogue Scale (1-10 cm) and was categorized as: No pain, Mild pain, Moderate pain, Severe pain based on the scale.

Statistical analysis

Continuous variables were expressed as mean ± standard deviation (SD). Categorical variables were presented as frequencies and percentages. The chi-square test was used for association analysis. Student’s t-test was used for group comparisons. A p-value less than 0.05 was considered significant. Analysis was performed using IBM SPSS Version 26 (IBM Corp., Armonk, NY, USA). Pearson correlation was applied while correlating DAS 28 score with VAS Score, and point biserial correlation was applied while correlating DAS-28 score with clinical outcome of sleep quality (categorical variable) and insomnia (Categorical variable). For cut-off value determination, the Receiver Operating Characteristic (ROC) was applied.

Ethical consideration

The study was approved by the Institutional Ethics Committee (Approval No. KIIT/KIMS/IEC/1169/2023) prior to the recruitment of participants.

## Results

The study included 176 subjects in total, comprising 88 RA patients in the case group and 88 healthy controls who were matched for sex and age. The demographic profiles of the participants are displayed in Table 1.

**Table 1:** Socio-demographic profile with co-morbidities of RA patients with comparison to healthy control t-test was applied for continuous data comparison. Chi-square test was applied for categorical data comparison.

Variables	RA Patients (N=88)	Controls (N=88)	t-value/chi-square value	p-value
Age (Mean ± SD)	52.30 ± 11.57	51.78 ± 8.84	0.33	0.742
Gender				
Female	72 (81.8%)	55 (62.5%)	9.05	0.04
Male	16 (18.2%)	33 (37.5%)		
Socioeconomic Status (SES)				
Low	41 (46.6%)	41 (46.6%)	0.0	1.00
Middle	47 (53.4%)	47 (53.4%)		
Co-morbidities				
Chronic Kidney Disease (CKD)	1 (1.14%)	1 (1.14%)	0.0	1.00
Hypertension	34 (38.6%)	22 (25.0%)	0.25	0.345
Diabetes Mellitus (DM)	24 (27.3%)	13 (14.8%)	0.32	0.773
Hypothyroidism	3 (3.4%)	3 (3.4%)	0	1.00
Osteoarthritis	3 (3.4%)	3 (3.4%)	0	1.00

The mean age of RA patients was 52.30 ± 11.57 years, while for those in the control group it was 51.78 ± 8.84 years. The difference was not statistically significant (p = 0.742), indicating the age distributions among the two groups were the same. There were 72 (81.8%) female and 16 (18.2%) male RA patients. In gender distribution, female patients were significantly more in comparison to male patients (p-value: 0.04). Socioeconomic status (SES) was similar in both groups with no significant difference (p-value: 1.00).

Disease activity score (DAS-28) classification

**Figure 1:**
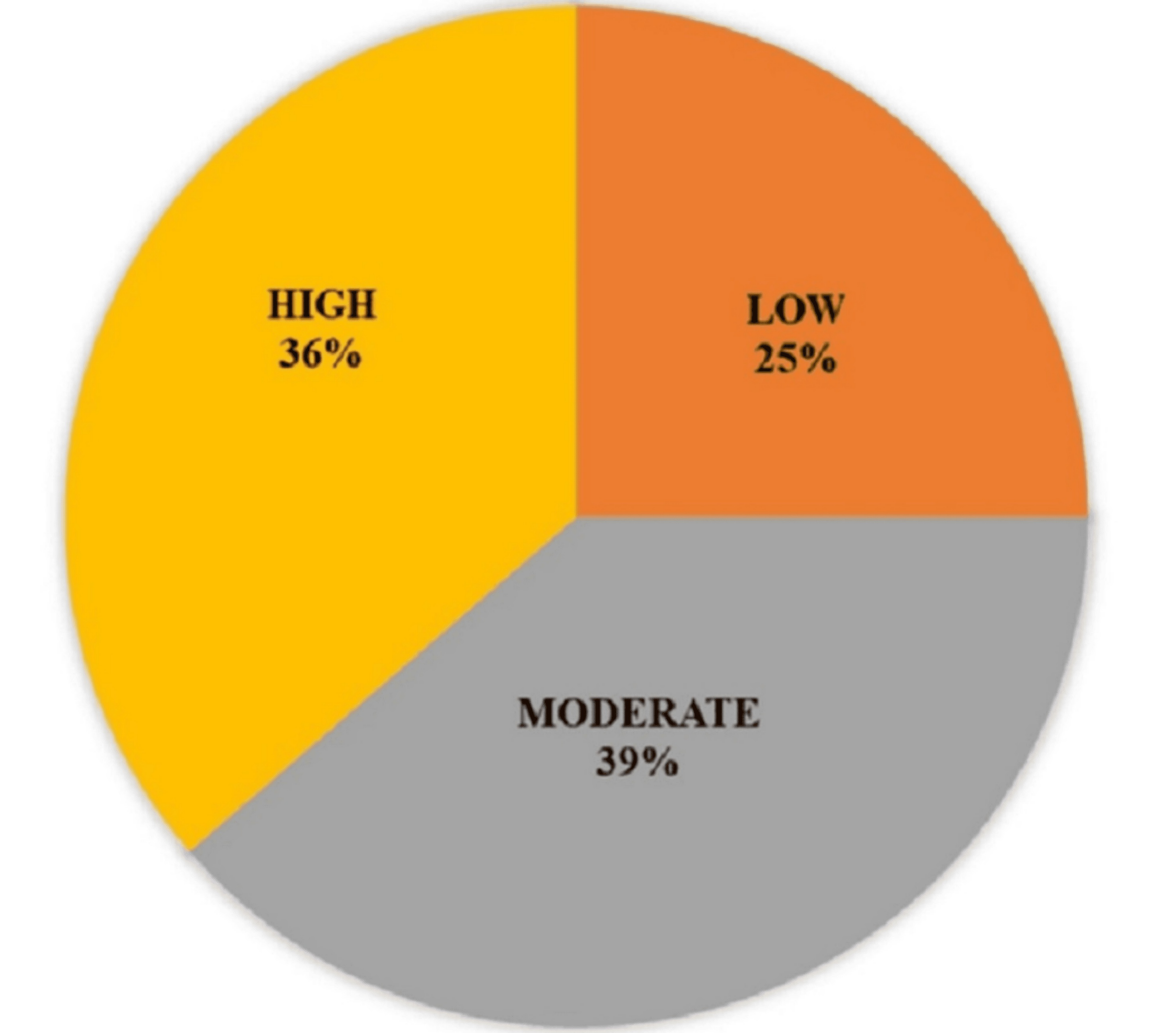
Distribution of RA Patients by DAS-28 Score DAS-28: Disease Activity Score-28

Figure 1 illustrates the classification of rheumatoid arthritis (RA) patients based on the Disease Activity Score (DAS-28). Disease activity was categorized as follows: severe (DAS-28 > 5.1), moderate (DAS-28 > 3.2 to ≤ 5.1), and mild (DAS-28 ≥ 2.6 and ≤ 3.2). Among RA patients, 22 (25.0%) had mild disease, 34 (38.6%) had moderate disease, and 32 (36.4%) had severe disease.

Comparison of pain perception, sleep quality, and insomnia

Table 2 explains a comparison of pain perception, sleep quality and insomnia between the RA group and healthy control group. RA patients reported significantly higher pain levels than controls (p < 0.001), with 26 (29.5%) experiencing severe pain, whereas none of the controls reported severe pain. Furthermore, RA had a substantial negative impact on sleep quality, as 66 (74.0%) of RA patients exhibited significantly poorer sleep quality compared to only 5 (5.7%) of controls (p < 0.001). Insomnia prevalence and severity, poor sleep quality, and pain perception were higher in rheumatoid arthritis (RA) patients than in healthy controls.

**Table 2:** Comparison of Pain Perception, Sleep Quality, and Insomnia Between RA Patients and Healthy Controls PSQI Score: Pittsburgh Sleep Quality Index Score ISI score: Insomnia Severity Index Score (The Insomnia Severity Index (ISI) is a scoring system (0-28) that evaluates an individual's perceived insomnia over the past two weeks by addressing both nighttime symptoms and the impact on daytime functioning).

Variables	RA Patients (N=88)	Controls (N=88)	Chi-Square (χ²)	p-value
Pain Perception Score (0-10 cm scale)			81.95	0.001
Mild (>0 to ≤3 cm)	22 (25.0%)	7 (8.0%)		
Moderate (>3 to ≤6 cm)	18 (20.5%)	1 (1.1%)		
Severe (>6 to ≤10 cm)	26 (29.5%)	0 (0.0%)		
No Pain	22 (25.0%)	80 (90.9%)		
Sleep Quality (PSQI Score)			84.99	0.001
Not Poor Sleeper (PSQI <5)	22 (25.0%)	83 (94.3%)		
Poor Sleeper (PSQI >5)	66 (74.0%)	5 (5.7%)		
Insomnia			87.46	0.001
Absent	21 (23.9%)	83 (94.3%)		
Present	67 (76.1%)	5 (5.7%)		
Insomnia Severity (Insomnia Severity Index)			98.05	0.001
Insignificant (0-7 score)	21 (23.9%)	83 (94.3%)		
Moderate (15-21 score)	37 (42.0%)	0 (0.0%)		
Severe (22-28 score)	24 (27.3%)	0 (0.0%)		
Subthreshold (8-14 score)	6 (6.8%)	5 (5.7%)		

Furthermore, a significantly higher prevalence of insomnia was observed among RA patients, with 67 (76.1%) experiencing sleep disturbances compared to only 5 (5.7%) of controls, indicating a strong association between RA and insomnia. In terms of insomnia severity, 37 (42.0%) of RA patients reported moderate insomnia, 24 (27.3%) had severe insomnia, and 6 (6.8%) experienced subthreshold insomnia. In contrast, 83 (94.3%) of controls had insignificant insomnia, with none reporting moderate or severe insomnia. These findings emphasize the substantial impact of RA on pain, sleep quality, and the burden of insomnia.

Correlation of disease severity of RA with pain, sleep quality and insomnia

A significant pain was observed in the RA study population as observed from the Visual Analogue Scale (VAS). Thus DAS-28 disease severity score was correlated with VAS score, sleep quality and insomnia individually. The correlation coefficient determined by Pearson Correlation analysis revealed a positive significant correlation between DAS-28 and VAS Score (r-value: 0.820, p-value: 0.00001). Similarly, the correlation of DAS-28 score with sleep quality and insomnia was determined by point biserial correlation analysis, which also showed a positive significant correlation (Table 3).

**Table 3:** Correlation of DAS-28 disease severity activity with VAS score, sleep quality and insomnia p-value <0.05: Significant (S) p-value was calculated from Pearson Correlation VAS: Visual Analogue Scale

Variables	DAS-28 disease severity (Correlation Coefficient, r-value)	Significance p-value
VAS score	0.8207	0.00001 (S)
Sleep quality	0.7643	0.00001 (S)
Insomnia	0.0707	0.00001 (S)

**Figure 2:**
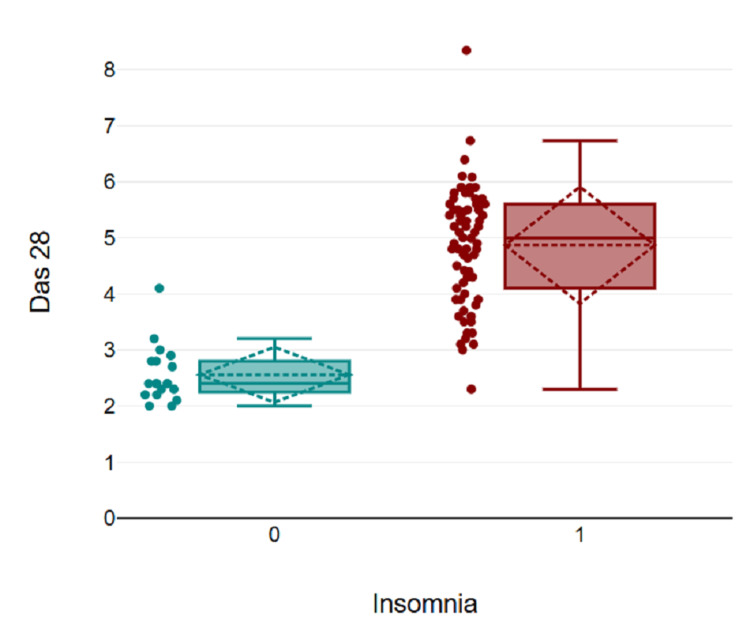
Correlation of DAS-28 with the incidence of insomnia x-axis: The incidence of insomnia (No=0, Yes=1) y-axis: Scores of DAS-28

Figure 2 shows a significant positive correlation between DAS-28 and insomnia. As the DAS-28 score increases, the incidence of insomnia also increases.

Prediction of insomnia based on DAS-28 in RA patients

**Figure 3:**
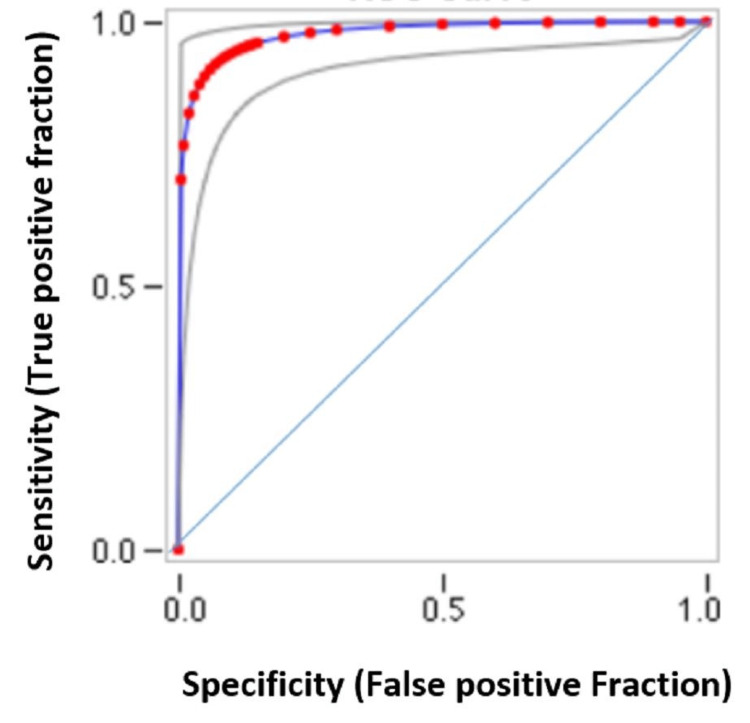
ROC of DAS-28 severity score for prediction of insomnia in RA patients ROC: Receiver Operating Characteristic ROC of DAS-28 severity score for prediction of insomnia in RA patients Red symbols and blue line shows the fitted ROC. Gray lines show the 95% confidence intervals of the fitted ROC. Blue line shows the reference line.

Disease Activity Score-28 (DAS-28) is a disease assessment tool through evaluation of inflammation and pain in 28 joints to monitor the disease severity in RA patients. As there is a strong positive correlation between pain, sleep quality and DAS-28 score, the chances of insomnia tend to be higher in patients with higher DAS-28 values. Based on Receiver Operating Characteristic (ROC) analysis, the cut-off value is determined from the Area Under the Curve (AUC) (Table 4). AUC of 0.974 indicates a high level of accuracy with 88% sensitivity for DAS-28 score in predicting insomnia (Figure 3), which means that 88% of individuals who were experiencing insomnia can be detected from the DAS-28 score. Those patients who do not have insomnia, the DAS-28 score can also be able to detect in 99% of individuals correctly. A DAS-28 score above 2.9 is predictive of poor sleep quality with insomnia in RA patients.

**Table 4:** ROC of DAS-28 disease severity with cut-off value for prediction of insomnia The main test statistic derived from the ROC curve is the AUC (Area Under the Curve).

	Mean ± SD	AUC	P-value	CI 95%	Sensitivity	Specificity	Cut-off
DAS-28 disease severity score	4.36 ± 1.35	0.974	0.00001	(0.8878-0.9952)	88%	99%	2.9

## Discussion

Among the 88 cases of RA studied a significant gender disparity was evident, with 72 (81.8%) females and 16 (18.2%) males resulting in a gender ratio of approximately 4.5:1. This pronounced predominance of females aligns with established epidemiological trends indicating higher susceptibility to RA among women as discussed by Smolen et al. and Sparks [3,10].

The age distribution analysis revealed that the majority (58.4%) of RA cases fell within the 40-59 years age group, indicating a peak incidence during middle adulthood. This finding corresponds with global epidemiological data showing that RA typically begins in middle age, as highlighted by Cross et al. and Eriksson et al. [11,12].

This demographic subgroup often faces additional challenges due to comorbidities and age-related complications, necessitating tailored therapeutic approaches. Statistical analysis indicated no significant association between age distribution and gender among RA cases (p=0.742). This suggests that while there is a distinct gender disparity in RA prevalence, the age distribution within each gender group follows a similar pattern. This result is in line with earlier studies of Sparks and Dessein et al., which found similar age distributions for men and women [10,13].

It is often acknowledged that rheumatoid arthritis (RA) sufferers frequently have sleep difficulties. Estimates of the prevalence of sleep problems in RA patients range from 30% to 70%, according to several studies [14,15]. Using the Pittsburgh Sleep Quality Index (PSQI), 66 (74.0%) of RA patients in our study were categorized as poor sleepers, which is substantially greater than the control group (5.7%, p<0.001). This result is consistent with earlier studies showing that chronic pain, inflammation, and the severity of the disease all lead to disturbed sleep architecture [5].

The relationship between RA and sleep disturbances is multifaceted, involving both physiological and psychological factors. Chronic pain, systemic inflammation, and the use of medications such as corticosteroids are significant contributors to poor sleep quality. Additionally, studies suggest that increased levels of pro-inflammatory cytokines, particularly tumor necrosis factor-alpha (TNF-α) and interleukin-6 (IL-6), may disrupt sleep patterns by affecting circadian rhythms and the central nervous system’s regulatory mechanisms [6].

The Disease Activity Score-28 (DAS-28) is commonly used to evaluate the variable degrees of disease activity that define RA. Of the RA patients in our study, 34 (38.6%) had moderate disease activity and 32 (36.4%) had high disease activity. This is in line with studies from around the world showing that despite treatment efforts, a sizable percentage of RA patients continue to have joint degeneration and inflammation [16].

It is commonly known that the severity of the condition affects day-to-day functioning. Increased joint pain, stiffness, and exhaustion are linked to high disease activity, and these factors ultimately lead to mobility issues and poor well-being. Moreover, severe RA cases often require more aggressive treatment strategies, including immunosuppressive therapy, which can have adverse effects on sleep patterns [4].

There is mounting evidence that the severity of RA disease and sleep difficulties are correlated. Patients in our study were more likely to report insomnia and poor sleep quality if their DAS-28 scores were greater (p<0.001). The literature has previously reported a correlation between greater disease activity and shorter sleep duration, higher fragmentation, and lower efficiency [7].

Pain perception plays a crucial role in this association. Our results indicate that cases with severe pain had significantly higher rates of insomnia (p<0.001), highlighting the role of pain in sleep disruption. This is supported by previous research indicating that poor sleep increases pain sensitivity, creating a vicious cycle of worsening RA symptoms and sleep disturbances [17]. Furthermore, increased inflammation due to disrupted sleep can exacerbate disease progression, emphasizing the need for a holistic approach in managing RA patients [18].

Two popular instruments for evaluating sleep disorders are the Insomnia Severity Index (ISI) and the Pittsburgh Sleep Quality Index (PSQI). In our study, 66 (74.0%) of RA patients had a PSQI score >5, indicating poor sleep quality, while 67 (76.1%) of RA patients had insomnia based on the ISI score. Among them, 37 (42.0%) had moderate insomnia and 24 (27.3%) had severe insomnia (p<0.001). These findings are consistent with prior studies that have used similar scoring tools to demonstrate the burden of sleep disturbances in RA patients [19, 20].

Numerous elements of sleep, such as length, latency, efficiency, and disruptions, are measured by the PSQI. According to earlier research, RA patients commonly experience non-restorative sleep, frequent awakenings, and trouble settling asleep, which can result in daytime weariness and cognitive impairment [19]. Similarly, the ISI allows for a more comprehensive evaluation of insomnia severity, which is particularly useful in understanding the impact of sleep disturbances on RA disease progression and quality of life. Given these findings, sleep assessment should be an integral part of RA management, and targeted interventions such as cognitive behavioral therapy for insomnia (CBT-I) or pharmacologic options should be considered [20].

Recommendation

The strong association between sleep disturbances and RA severity underscores the need for a multidisciplinary approach to patient management. Routine assessment of sleep quality should be incorporated into RA evaluation, as addressing sleep disturbances could potentially improve pain management, inflammation control, and overall disease outcomes. Plans for managing RA should take into account interventions such as targeted pharmaceutical therapies, lifestyle changes, and cognitive behavioral therapy for insomnia (CBT-I).

Limitation of the study was a smaller sample size, as the study was conducted in a single-centre setting. Additionally, the selection of study participants was strictly based on predefined inclusion and exclusion criteria, which limited the sample size.

## Conclusions

Poor sleep quality and sleep disorders contribute to inflammation and disease progression in RA patients, but many times these sleep disorders are overlooked during investigations, which significantly impacts overall quality of life. The study highlights the prevalence of sleep disorders in RA patients and determines the association of sleep disorders with disease activity. The findings indicate that progression of insomnia severity and decreased sleep quality were strongly correlated with higher RA disease activity as measured by the Disease Activity Score-28. Pain perception is a significant determinant that influences sleep quality; a higher pain score as measured by the Visual Analogue Scale (VAS) score significantly correlates with sleep disruption. This reinforces the cyclical relationship between pain, inflammation, and sleep impairment. Importantly, the DAS-28 assessment score may serve as a predictive tool for screening RA patients with sleep disorder (Insomnia), which could be a valuable adjunct to conventional treatment strategies, for potentially improving both physical and psychological well-being.
